# Distributed State Estimation with Unknown Inputs and Heavy-Tailed Noises

**DOI:** 10.3390/s26216835

**Published:** 2026-10-29

**Authors:** Hui Li, Xingye Wang, Shuang Ju

**Affiliations:** 1 The School of Electrical Engineering, Yanshan University, Qinhuangdao 066000, China; yandalihui@163.com (H.L.); wangxingye@stumail.ysu.edu.cn (X.W.); 2 The School of Electrical and Electronic Engineering, Shijiazhuang Tiedao University, Shijiazhuang 050043, China

**Keywords:** distributed filter, unknown inputs, heavy-tailed noises, stability, sensor networks

## Abstract

This paper addresses the distributed filtering problem for linear systems subject to unknown inputs and heavy-tailed measurement noises. The unknown inputs are modeled using a non-informative prior distribution, while the heavy-tailed noises are modeled using a Student’s t distribution. Leveraging variational Bayesian methods, a local filter is designed to jointly estimate system states and unknown inputs in the presence of heavy-tailed measurement noises. Subsequently, a distributed filtering framework is proposed by integrating the designed local filter with the consensus on information method. Moreover, a sufficient condition is derived to ensure the boundedness of the estimation error covariance of the proposed distributed filter. Finally, simulation results from a target tracking system are presented to demonstrate the effectiveness of the proposed distributed filtering approach.

## 1. Introduction

In the past three decades, wireless sensor networks (WSNs) have been widely applied in areas such as power systems [1], cooperative localization [2], and target tracking [3]. Distributed state estimation or filtering is a fundamental problem in WSNs [4]. In this context, each sensor cooperatively estimates the state using its local measurements and information received from neighboring sensors. Distributed state estimation has been extensively studied due to its advantages in terms of low computational and communication overhead and high scalability. Some typical algorithms include Kalman consensus filters [5,6], distributed recursive filters [7], diffusion distributed Kalman filters [8], and distributed set-membership filters [9,10]. These previous studies have proposed various effective methods for distributed state estimation. Nevertheless, most of the existing methods fail to consider unknown inputs.

In practical scenarios, it is often inevitable for systems to encounter unknown inputs, such as the unmeasured wind resistance of aircraft and the field voltage in brushless excitation systems [11]. As a result, state estimation in the presence of unknown inputs has drawn significant attention. However, most existing research focuses on single-sensor or centralized models. Typical algorithms include the state augmentation-based Kalman filter (ASKF) [12], the unbiased minimum-variance (UMV) estimator [13], and the modified Kalman filter with a non-informative prior distribution [14,15]. Regarding distributed state estimation, two distributed filters were investigated in [16,17], where the filter gains were determined by the unbiased minimum variance criterion. In [18], an event-triggered distributed fusion algorithm was proposed for nonlinear systems with random sensor failures and unknown inputs, and the boundedness of the estimation error covariance was analyzed. In [19], a distributed filter was designed using Bayesian methods and a non-informative prior distribution under random link failures, and two sufficient conditions for stability were provided. Notably, the above-mentioned studies assume Gaussian noise. Consequently, they may not yield satisfactory estimation performance when dealing with heavy-tailed noise disturbances.

In practice, the measurement process may be affected by outliers caused by sensor failures or disturbances during wireless data acquisition and transmission, leading to a heavy-tailed distribution of measurement noise [20,21]. For example, field PMU data from the Western Electricity Coordinating Council (WECC) system have shown that PMU measurement errors can exhibit heavy-tailed non-Gaussian characteristics [22]. A recent study based on 48 h real-world GNSS measurements found clear heavy-tailed characteristics in the measurement errors, and the Student’s t distribution provided a better fit than the Gaussian distribution [23]. To ensure estimation performance in the presence of heavy-tailed noises, numerous robust filters have been developed, including Huber filters [24], maximum correntropy filters [25], minimum error entropy filters [26], particle filters [27], and Student’s t filters [28]. For a linear system with unknown inputs and heavy-tailed noises, a robust filter was designed using variational Bayesian methods in [29]. Note that the filter in [29] assumes that the unknown input follows a time-evolution model, which may lead to performance degradation when the unknown input changes abruptly. In [30], a filter was proposed for a nonlinear system with unknown inputs and heavy-tailed noises, relying on statistical linearization and the maximum correntropy criterion. The method presented in [30] is based on a single-sensor or centralized model, and the corresponding stability analysis remains unaddressed. Recently, robust signal processing has also attracted attention in industrial condition monitoring. For example, Gai et al. [31] proposed a fractional-order stochastic resonance method to enhance weak multi-frequency fault signals under strong background noise. In industrial sensor networks, unknown inputs and measurement outliers may also affect state estimation performance. Therefore, robust distributed state estimation could provide useful information for subsequent fault detection and diagnosis.

To further clarify the research gap, Table 1 summarizes existing centralized and distributed filters for systems with unknown inputs. However, distributed state estimation under both unknown inputs and heavy-tailed measurement noises remains insufficiently studied. In fact, unknown inputs represent uncertainties within the system process model, while heavy-tailed measurement noises signify uncertainties in the measurement process. Obviously, these two types of uncertainty can coexist in a practical system due to factors such as sensor faults, modeling errors, and environmental changes [29]. Inspired by the above analysis, this paper focuses on the problem of distributed state estimation in the presence of unknown inputs and heavy-tailed measurement noises. The main contributions are summarized as follows:

- Based on variational Bayesian inference and the consensus on information method, a distributed filter is designed for systems with unknown inputs and heavy-tailed measurement noises. Compared with the method in [29], the unknown inputs are modeled using a non-informative prior distribution, which enables the filter to mitigate the influence of different types of unknown inputs (see Theorem 2). Although the unknown inputs are also assumed to be completely unknown in [30], the filter requires that the system model and the measurement model are disturbed by the same unknown inputs. If the measurement model does not contain unknown inputs ($D_{k}=0$), the filter will not be able to update.
- The stability of the proposed distributed filter is investigated, and a sufficient condition is derived to ensure the boundedness of the estimation error covariance. Although the stability of distributed filters has been analyzed in [16,17,18], they do not establish a relationship between stability and uniform complete observability, and differences in design methods make it difficult to directly extend the existing methods. The stability condition in [19] requires the boundedness of matrix $L_{k}$ (see Lemma 1 in [19]), which is obtained by QR decomposition. The filter in this paper does not require QR decomposition. Therefore, the boundedness condition on $L_{k}$ is no longer required.

## 2. Background and Problem Formulation

**Figure 1:**
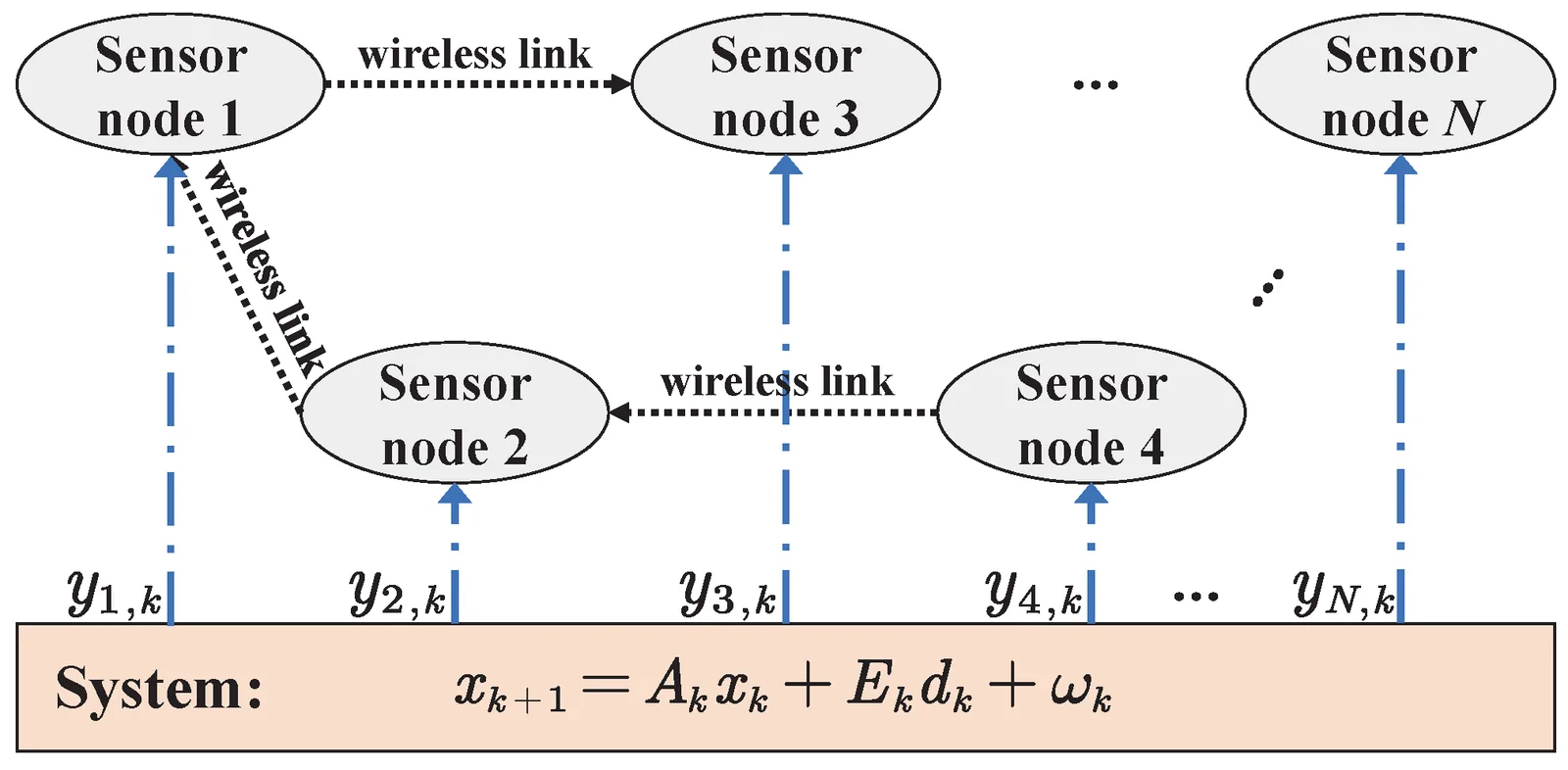
Distributed state estimation process over sensor networks.

This paper studies the distributed state estimation problem for a linear system over sensor networks. The state estimation process is illustrated by the example depicted in Figure 1. The target system is monitored by *N* sensor nodes. Each node measures the system state $x_{k}$ using its local sensor, generating the measurement output $y_{i,k}$. The system dynamics are influenced by an unknown input $d_{k}$, and sensors may be disrupted by unforeseen external disturbances, leading to heavy-tailed measurement noises. The sensor nodes are interconnected via wireless networks, enabling them to exchange information with neighboring nodes and collaboratively perform state estimation. The goal is to design a distributed algorithm for each node, allowing it to achieve accurate state estimation using its own measurement output $y_{i,k}$ and data received from other nodes.

The target system is modeled as follows:

(1) $$\;x_{k+1}=A_{k}x_{k}+E_{k}d_{k}+\omega_{k},$$

where $x_{k}\in R^{n_{x}}$ is the system state, $A_{k}\in R^{n_{x}\times n_{x}}$ is the system matrix, $d_{k}\in R^{p}$ is the unknown input, $E_{k}\in R^{n_{x}\times p}$ is the known matrix, $\omega_{k}\in R^{n_{x}}$ is zero-mean Gaussian noise with covariance $Q_{k}$. Consistent with the assumptions in [13,19], we assume that $\mathrm{rank}(E_{k})=p$. This rank condition implies that $p<n_{x}$, indicating that the number of states exceeds the number of unknown inputs. For notational simplicity, the same symbols are used for random quantities and their realizations unless otherwise specified. In probability density functions and integrals, these symbols denote the possible values of the corresponding quantities. According to [14], we model $d_{k}$ using a non-informative prior distribution:

(2) $$\;p(d_{k})\propto 1.$$

This non-informative prior indicates that no particular value of $d_{k}$ is preferred, reflecting the lack of prior information about the unknown input.

The sensor network consists of *N* sensor nodes. The measurement equation of the sensor node *i* is

(3) $$\;y_{i,k}=C_{i,k}x_{k}+\varepsilon_{i,k},$$

where i∈V={1,2,…,N}. The measurement output of the *i*-th sensor node is denoted by $y_{i,k}\in R^{n_{y}}$, and $C_{i,k}\in R^{n_{y}\times n_{x}}$ represents the measurement matrix. The heavy-tailed measurement noise $\varepsilon_{i,k}$ is modeled using a Student’s t distribution, as described below:

(4) $$\;p\left(\varepsilon_{i,k}\right)=\mathrm{St}\left(\varepsilon_{i,k};0,R_{i,k},\nu_{i,k}\right)$$

where $\mathrm{St}\left(\varepsilon_{i,k};0,R_{i,k},\nu_{i,k}\right)$ denotes the Student’s t PDF with mean 0, scale matrix $R_{i,k}$, and degrees of freedom (DOFs) $\nu_{i,k}$. Using Equations (3) and (4), the likelihood PDF $p(y_{i,k}|x_{k})$ can be formulated as

$$\begin{array}{ccc} & & \;p\left(y_{i,k}|x_{k}\right)=\mathrm{St}\left(y_{i,k};C_{i,k}x_{k},R_{i,k},\nu_{i,k}\right). \end{array}$$

According to [32], we assume that the posterior PDF $p(x_{k-1}|y_{i,1:k-1})$ follows a Gaussian distribution

(5) $$\begin{array}{ccc} & & \;p\left(x_{k-1}|y_{i,1:k-1}\right)=N\left(x_{k-1};{\hat{x}}_{i,k-1},P_{i,k-1}\right) \end{array}$$

where ${\hat{x}}_{i,k-1}$ is the posterior state estimate of node *i*, and $P_{i,k-1}$ is the corresponding covariance. Since the likelihood PDF $p(y_{i,k}|x_{k})$ follows a Student’s t distribution rather than a Gaussian distribution, it is difficult to obtain a closed-form posterior PDF $p(x_{k}|y_{i,1:k})$ by Bayes’ theorem. In general, $p(x_{k}|y_{i,1:k})$ is not Gaussian. Since the Student’s *t* PDF can be represented as an infinite mixture of Gaussian PDFs, we introduce an auxiliary random variable $\Lambda_{i,k}$ following a Gamma distribution, and use $\lambda_{i,k}$ to denote its possible value. Then, the likelihood PDF $p(y_{i,k}|x_{k})$ can be rewritten as follows [28]:

(6) $$p\left(y_{i,k}|x_{k}\right)=\mathrm{St}\left(y_{i,k};C_{i,k}x_{k},R_{i,k},\nu_{i,k}\right)=\int N\left(y_{i,k};C_{i,k}x_{k},R_{i,k}/\lambda_{i,k}\right)G\left(\lambda_{i,k};\frac{\nu_{i,k}}{2},\frac{\nu_{i,k}}{2}\right)d\lambda_{i,k},$$

where G(·;α,β) denotes the Gamma PDF with shape parameter α and rate parameter β. According to Equation (6), the likelihood PDF $p\left(y_{i,k}|x_{k}\right)$ can be written as the following hierarchical Gaussian form:

(7) $$\begin{array}{ccc} & & \;p\left(y_{i,k}|x_{k},\lambda_{i,k}\right)=N\left(y_{i,k};C_{i,k}x_{k},R_{i,k}/\lambda_{i,k}\right), \end{array}$$

(8) $$\begin{array}{ccc} & & \;p_{\Lambda_{i,k}}\left(\lambda_{i,k}\right)=G\left(\lambda_{i,k};\frac{\nu_{i,k}}{2},\frac{\nu_{i,k}}{2}\right). \end{array}$$

Based on Equations (5), (7), and (8), we can obtain an approximate posterior PDF $q(x_{k})$ in Gaussian form using the variational Bayesian method.

**Remark** **1.** *The Student’s t distribution is adopted for two main reasons. First, its Gaussian-Gamma scale-mixture representation facilitates the derivation of the proposed variational Bayesian filter. Second, the Student’s t distribution has shown practical value in state estimation and multi-sensor fusion. For example, it has been applied to cooperative navigation of autonomous underwater vehicles [33], multi-sensor fusion for autonomous vehicle localization [34], and state estimation in power systems [35]. In addition, the proposed framework may also provide a basis for exploring other heavy-tailed noise models, such as the Laplace distribution [36], although such an extension would require appropriate modifications to the probabilistic model and a re-derivation of the corresponding Bayesian update equations.*

**Remark** **2.** *In this paper, the DOF parameter of the Student’s t distribution is treated as a fixed model parameter. In practice, it can be determined offline using representative sensor measurement data. For example, the maximum likelihood method can be used to estimate a suitable DOF value [37]. Alternatively, the DOF can be modeled as a Gamma-distributed random variable and estimated using variational Bayesian methods [38]. However, this approach introduces additional online computational costs. Considering the limited computational resources of sensor nodes, we treat the DOF as a fixed parameter in this paper.*

The communication topology of the wireless sensor network is represented by a directed graph G(V,E,A), with the node set V={1,2,…,N}, the edge set E⊆V×V, and the weighted matrix $A={\left[a_{i,j}\right]}_{N\times N}$. Matrix A is considered to be row stochastic, i.e., $\sum_{j\in V}a_{i,j}=1$. All elements of matrix A are nonnegative, and $a_{i,i}>0$ for all i∈V. An edge (i,j) belongs to E if and only if $a_{i,j}>0$. This means that node *i* can directly receive information from node *j*. In this case, node *j* is an in-neighbor of *i*, and node *i* is an out-neighbor of *j*. The set consisting of node *i* and all its in-neighbors is denoted by $N_{i}=\left\{i\right\}⋃\left\{j\in V|\left(i,j\right)\in E\right\}$.

## 3. Distributed Filter Design

The distributed filter is composed of two main parts. The first part is the local filter, which utilizes the measurement outputs $y_{i,k}$ to generate the local state estimate ${\hat{x}}_{i,k}$ and its corresponding error covariance $P_{i,k}$. During this local filtering process, each node operates autonomously and does not require communication with other nodes. The second part of the distributed filter involves the exchange of ${\hat{x}}_{i,k}$ and $P_{i,k}$ with neighboring nodes $j\in N_{i}$ through wireless networks, following a consensus strategy. This collaborative step enables nodes to share information and improve the overall estimation performance. We first present the design of the local filter.

### 3.1. Local Filter with Unknown Inputs and Heavy-Tailed Noises

In this subsection, we present a local filter using the variational Bayesian method. The proposed local filter is described in Steps 1–2, and the detailed design process is provided in the Appendix A.

**Step 1:** State prediction

Let ${\hat{x}}_{i,k|k-1}$ denote the state prediction, and let $P_{i,k|k-1}$ denote the predicted error covariance. Sensor node *i* calculates ${\hat{x}}_{i,k|k-1}$ and $P_{i,k|k-1}$ as follows:

(9) $$\begin{array}{ccc} & & \;{\hat{x}}_{i,k|k-1}=A_{k-1}{\hat{x}}_{i,k-1}, \end{array}$$

(10) $$\begin{array}{ccc} & & \;P_{i,k|k-1}=A_{k-1}P_{i,k-1}A_{k-1}^{T}+Q_{k-1}. \end{array}$$

**Step 2:** Filtering

Let ${\hat{x}}_{i,k}$ and $P_{i,k}$ denote the posterior state estimate and its corresponding error covariance, respectively. Based on the variational Bayesian approximation process, ${\hat{x}}_{i,k}$ and $P_{i,k}$ are obtained by the following iterative procedure.

(i) Initialize the iteration parameters as follows:

(11) $$\begin{array}{ccc} & & \;\alpha_{i,k}^{\left(0\right)}=\alpha_{0},\;\beta_{i,k}^{\left(0\right)}=\beta_{0}. \end{array}$$

(ii) Perform the following *L* fixed-point iterations (l=0,1,…,L−1):

(12) $$\begin{array}{ccc} & & \;{\hat{\lambda }}_{i,k}^{\left(l+1\right)}=\frac{\alpha_{i,k}^{\left(l\right)}}{\beta_{i,k}^{\left(l\right)}},\;{\tilde{R}}_{i,k}^{\left(l+1\right)}=\frac{R_{i,k}}{{\hat{\lambda }}_{i,k}^{\left(l+1\right)}}, \end{array}$$

(13) $$\begin{array}{ccc} & & \;{\overset{˘}{R}}_{i,k}^{\left(l+1\right)}={\tilde{R}}_{i,k}^{\left(l+1\right)}+C_{i,k}P_{i,k|k-1}C_{i,k}^{T}, \end{array}$$

(14) $$\begin{array}{ccc} & & \;P_{i,k}^{dd,\left(l+1\right)}={\left[{\left(C_{i,k}E_{k-1}\right)}^{T}{\left({\overset{˘}{R}}_{i,k}^{\left(l+1\right)}\right)}^{-1}C_{i,k}E_{k-1}\right]}^{-1}, \end{array}$$

(15) $$\begin{array}{ccc} & & \;{\hat{d}}_{i,k-1}^{\left(l+1\right)}=P_{i,k}^{dd,\left(l+1\right)}{\left(E_{k-1}\right)}^{T}{\left(C_{i,k}\right)}^{T}{\left({\overset{˘}{R}}_{i,k}^{\left(l+1\right)}\right)}^{-1}\left(y_{i,k}-C_{i,k}{\hat{x}}_{i,k|k-1}\right), \end{array}$$

(16) $$\begin{array}{ccc} & & \;P_{i,k}^{xd,\left(l+1\right)}=\left[E_{k-1}-P_{i,k|k-1}C_{i,k}^{T}{\left({\overset{˘}{R}}_{i,k}^{\left(l+1\right)}\right)}^{-1}C_{i,k}E_{k-1}\right]P_{i,k}^{dd,\left(l+1\right)}, \end{array}$$

(17) $$\begin{array}{ccc} & & \;{\hat{x}}_{i,k}^{\left(l+1\right)}={\hat{x}}_{i,k|k-1}+P_{i,k}^{xd,\left(l+1\right)}{\left(P_{i,k}^{dd,\left(l+1\right)}\right)}^{-1}{\hat{d}}_{i,k-1}^{\left(l+1\right)}\\ & & \;+P_{i,k|k-1}C_{i,k}^{T}{\left({\overset{˘}{R}}_{i,k}^{\left(l+1\right)}\right)}^{-1}\left(y_{i,k}-C_{i,k}{\hat{x}}_{i,k|k-1}\right), \end{array}$$

(18) $$\begin{array}{ccc} & & \;P_{i,k}^{\left(l+1\right)}=P_{i,k|k-1}-P_{i,k|k-1}C_{i,k}^{T}{\left({\overset{˘}{R}}_{i,k}^{\left(l+1\right)}\right)}^{-1}C_{i,k}P_{i,k|k-1}\\ & & \;+P_{i,k}^{xd,\left(l+1\right)}{\left(P_{i,k}^{dd,\left(l+1\right)}\right)}^{-1}{\left(P_{i,k}^{xd,\left(l+1\right)}\right)}^{T}, \end{array}$$

(19) $$\begin{array}{ccc} & & \;\Delta_{i,k}^{\left(l+1\right)}=\left(y_{i,k}-C_{i,k}{\hat{x}}_{i,k}^{\left(l+1\right)}\right){\left(y_{i,k}-C_{i,k}{\hat{x}}_{i,k}^{\left(l+1\right)}\right)}^{T}+C_{i,k}P_{i,k}^{\left(l+1\right)}C_{i,k}^{T}, \end{array}$$

(20) $$\begin{array}{ccc} & & \;\alpha_{i,k}^{\left(l+1\right)}=\frac{\nu_{i,k}+n_{y}}{2},\;\beta_{i,k}^{\left(l+1\right)}=\frac{1}{2}\left[\mathrm{tr}\left(\Delta_{i,k}^{\left(l+1\right)}R_{i,k}^{-1}\right)+\nu_{i,k}\right]. \end{array}$$

(iii) Let ${\hat{x}}_{i,k}={\hat{x}}_{i,k}^{\left(L\right)}$ and $P_{i,k}=P_{i,k}^{\left(L\right)}$.

### 3.2. Distributed Filtering via Information Consensus

In this subsection, we extend the local filtering approach to the distributed setting. The distributed filtering process comprises three steps. Steps 1–2 of the distributed filter are identical to those of the local filter, while Step 3 is described as follows:

**Step 3:** Consensus

Following the consensus on information method and the information filtering framework in Section 3.1 of [5], let $\delta_{i,k}$ and ${Ω}_{i,k}$ denote the local information vector and the local information matrix, respectively. They are calculated as $\delta_{i,k}={\left(P_{i,k}^{\left(L\right)}\right)}^{-1}{\hat{x}}_{i,k}^{\left(L\right)}$ and ${Ω}_{i,k}={\left(P_{i,k}^{\left(L\right)}\right)}^{-1}$. In this step, each node exchanges $\delta_{i,k}$ and ${Ω}_{i,k}$ with the other nodes through $T_{c}$ consensus iterations to cooperatively estimate the system state.

(i) Initialize the consensus variables as follows:

(21) $$\begin{array}{ccc} & & \;\delta_{i,k}\left(0\right)={\left(P_{i,k}\right)}^{-1}{\hat{x}}_{i,k},\;{Ω}_{i,k}\left(0\right)={\left(P_{i,k}\right)}^{-1}, \end{array}$$

(ii) Perform the following $T_{c}$ steps of consensus ($t=0,1,\ldots ,T_{c}-1$):

Send $\left\{\delta_{i,k}\left(t\right),{Ω}_{i,k}\left(t\right)\right\}$ to the out-neighbors, and collect $\left\{\delta_{j,k}\left(t\right),{Ω}_{j,k}\left(t\right)\right\}$, $j\in N_{i}\backslash \left\{i\right\}$ from the in-neighbors.

Calculate $\delta_{i,k}\left(t+1\right)$ and ${Ω}_{i,k}\left(t+1\right)$ by

(22) $$\begin{array}{ccc} & & \;\delta_{i,k}\left(t+1\right)=\sum_{j\in N_{i}}a_{i,j}\delta_{j,k}\left(t\right), \end{array}$$

(23) $$\begin{array}{ccc} & & \;{Ω}_{i,k}\left(t+1\right)=\sum_{j\in N_{i}}a_{i,j}{Ω}_{j,k}\left(t\right). \end{array}$$

(iii) Calculate ${\hat{x}}_{i,k}$ and $P_{i,k}$ by

(24) $$\begin{array}{ccc} & & \;{\hat{x}}_{i,k}={\left[{Ω}_{i,k}\left(T_{c}\right)\right]}^{-1}\delta_{i,k}\left(T_{c}\right), \end{array}$$

(25) $$\begin{array}{ccc} & & \;P_{i,k}={\left[{Ω}_{i,k}\left(T_{c}\right)\right]}^{-1}. \end{array}$$

The implementation steps of the proposed distributed filter are summarized in Algorithm 1.
**Algorithm 1** Distributed Filter Algorithm**Initialization:** Begin with ${\hat{x}}_{i,0}=x_{0}$, $P_{i,0}=P_{0}$.**Inputs:** ${\hat{x}}_{i,k-1}$, $P_{i,k-1}$, $\nu_{i,k}$, $n_{y}$, $A_{k-1}$, $Q_{k-1}$, $C_{i,k}$, $R_{i,k}$, $y_{i,k}$, *L*, $T_{c}$**Step 1. State prediction:**Calculate ${\hat{x}}_{i,k|k-1}$ by Equation (9), $P_{i,k|k-1}$ by Equation (10).**Step 2. Filtering:**Calculate the initial values $\alpha_{i,k}^{\left(0\right)}$ and $\beta_{i,k}^{\left(0\right)}$ by Equation (11).**for** l=0,…,L−1 **do**   Update ${\hat{\lambda }}_{i,k}^{\left(l+1\right)}$ and ${\tilde{R}}_{i,k}^{\left(l+1\right)}$ by Equation (12).   Update ${\overset{˘}{R}}_{i,k}^{\left(l+1\right)}$ by Equation (13), $P_{i,k}^{dd,\left(l+1\right)}$ by Equation (14).   Update ${\hat{d}}_{i,k-1}^{\left(l+1\right)}$ by Equation (15), $P_{i,k}^{xd,\left(l+1\right)}$ by Equation (16).   Update ${\hat{x}}_{i,k}^{\left(l+1\right)}$ by Equation (17), $P_{i,k}^{\left(l+1\right)}$ by Equation (18).   Update $\Delta_{i,k}^{\left(l+1\right)}$ by Equation (19), $\alpha_{i,k}^{\left(l+1\right)}$ and $\beta_{i,k}^{\left(l+1\right)}$ by Equation (20).**end for****Step 3. Consensus:**Calculate the initial values $\delta_{i,k}\left(0\right)$ and ${Ω}_{i,k}\left(0\right)$ by Equation (21).**for** $t=0,\ldots ,T_{c}-1$ **do**   Send $\left\{\delta_{i,k}\left(t\right),{Ω}_{i,k}\left(t\right)\right\}$ to out-neighbors, collect $\left\{\delta_{j,k}\left(t\right),{Ω}_{j,k}\left(t\right)\right\}$, $j\in N_{i}\backslash \left\{i\right\}$ from in-neighbors.   Update $\delta_{i,k}\left(t+1\right)$ by Equation (22), ${Ω}_{i,k}\left(t+1\right)$ by Equation (23).**end for**Calculate ${\hat{x}}_{i,k}$ by Equation (24), $P_{i,k}$ by Equation (25).**Outputs:** ${\hat{x}}_{i,k}$, $P_{i,k}$

## 4. Stability of the Distributed Filter

This section discusses the stability of the proposed distributed filter. Before presenting the key results, we introduce two definitions and a lemma. These will serve as the foundation for the subsequent analysis.

**Definition** **1** ([39])**.** *A matrix sequence pair $\left[A_{k},C_{k}\right]$, with $A_{k}\in R^{n\times n}$ and $C_{k}\in R^{m\times n}$ for k=0,1,2,…, is uniformly completely observable if there exist two positive constants $\alpha_{2}>\alpha_{1}>0$ and a positive integer h such that for all k=0,1,2,…*

$$\begin{array}{ccc} & & \;\alpha_{1}I_{n}\leq \sum_{j=k}^{k+h-1}\Phi_{j|k}^{T}C_{j}^{T}C_{j}\Phi_{j|k}\leq \alpha_{2}I_{n}. \end{array}$$

**Definition** **2** ([39])**.** *A matrix sequence pair $\left[A_{k},B_{k}\right]$, with $A_{k}\in R^{n\times n}$ and $B_{k}\in R^{n\times p}$ for k=0,1,2,…, is uniformly completely controllable if there exist two positive constants $\beta_{2}>\beta_{1}>0$ and a positive integer h such that for all k=h,h+1,…*

$$\begin{array}{ccc} & & \;\beta_{1}I_{n}\leq \sum_{j=k-h+1}^{k}\Phi_{k+1|j+1}B_{j}B_{j}^{T}\Phi_{k+1|j+1}^{T}\leq \beta_{2}I_{n}. \end{array}$$

**Lemma** **1** ([40])**.** *Given matrices $\Phi \in R^{m\times n}$, $\Sigma \in R^{m\times m}$, $Ω\in R^{m\times q}$, $E\in R^{n\times q}$, where 0<q≤m≤n,* Σ *is a symmetric positive-definite matrix, and* Ω *has full column rank. For any matrix $L\in R^{n\times m}$ satisfying the constraint LΩ=E, the following inequality holds:*

$$\begin{array}{ccc} & & \;L\Sigma L^{T}-L\Psi -\Psi^{T}L^{T}\geq L_{*}\Sigma L_{*}^{T}-L_{*}\Psi -\Psi^{T}L_{*}^{T}, \end{array}$$

*where*

$$\begin{array}{ccc} & & \;L_{*}=\Psi^{T}\Sigma^{-1}+\left(E-\Psi^{T}\Sigma^{-1}Ω\right){\left({Ω}^{T}\Sigma^{-1}Ω\right)}^{-1}{Ω}^{T}\Sigma^{-1} \end{array}$$

and $L_{*}Ω=E$.

To facilitate the stability analysis, we introduce an auxiliary stochastic system:

(26) $$\begin{array}{ccc} & & \;{\bar{x}}_{k+1}={\bar{A}}_{i,k}{\bar{x}}_{k}+{\bar{w}}_{k}, \end{array}$$

(27) $$\begin{array}{ccc} & & \;{\bar{y}}_{i,k}=C_{i,k}{\bar{x}}_{k}+{\bar{v}}_{k}, \end{array}$$

where i∈V, $\mathrm{Cov}({\bar{w}}_{k})=I$, $\mathrm{Cov}({\bar{v}}_{k})=I$, and

$$\begin{array}{ccc} & & \;{\bar{A}}_{i,k}=\left(I-G_{i,k+1}C_{i,k+1}\right)\frac{1}{\sqrt{a_{i,i}\left(T_{c}\right)}}A_{k},\\ & & \;G_{i,k}=E_{k-1}{\left[{\left(C_{i,k}E_{k-1}\right)}^{T}\left(C_{i,k}E_{k-1}\right)\right]}^{-1}{\left(C_{i,k}E_{k-1}\right)}^{T}. \end{array}$$

A classical Kalman filter can be constructed for the auxiliary stochastic system Equations (26) and (27):

(28) $$\begin{array}{ccc} & & \;{\bar{P}}_{k|k-1}={\bar{A}}_{i,k-1}{\bar{P}}_{k-1|k-1}{\bar{A}}_{i,k-1}^{T}+I, \end{array}$$

(29) $$\begin{array}{ccc} & & \;{\bar{K}}_{k}={\bar{P}}_{k|k-1}C_{i,k}^{T}{\bar{\Sigma }}_{k}^{-1},\;{\bar{\Sigma }}_{k}=C_{i,k}{\bar{P}}_{k|k-1}C_{i,k}^{T}+I, \end{array}$$

(30) $$\begin{array}{ccc} & & \;{\bar{P}}_{k|k}=\left(I-{\bar{K}}_{k}C_{i,k}\right){\bar{P}}_{k|k-1}{\left(I-{\bar{K}}_{k}C_{i,k}\right)}^{T}+{\bar{K}}_{k}{\bar{K}}_{k}^{T}, \end{array}$$

(31) $$\begin{array}{ccc} & & \;{\hat{x}}_{k|k-1}={\bar{A}}_{i,k-1}{\hat{x}}_{k-1|k-1}, \end{array}$$

(32) $$\begin{array}{ccc} & & \;{\hat{x}}_{k|k}={\hat{x}}_{k|k-1}+{\bar{K}}_{k}\left({\bar{y}}_{i,k}-C_{i,k}{\hat{x}}_{k|k-1}\right). \end{array}$$

**Theorem** **1.** *Consider the distributed filter given in Algorithm 1. There exists a positive constant $\bar{p}$ such that $P_{i,k}^{\left(L\right)}\leq \bar{p}I$ holds for any i∈V if the following conditions hold:*

*(i)*  *Given any i∈V, the matrix sequence pair $\left[{\bar{A}}_{i,k},C_{i,k}\right]$ is uniformly completely observable.*
*(ii)*  *$A_{k}$, $E_{k}$, $C_{i,k}$, $Q_{k}$, and $R_{i,k}$ are bounded matrix sequences for all k≥0 and any i∈V.*
*(iii)*  *The inverse matrix ${\left({\tilde{R}}_{i,k}^{\left(L\right)}\right)}^{-1}$ exists and is bounded for all k≥0 and any i∈V.*
*(iv)*  *The matrix product $C_{i,k+1}E_{k}$ has full column rank and a bounded Moore–Penrose inverse for all k≥0 and any i∈V.*
*(v)*  *The initial error covariance $P_{0}$ is bounded.*

**Proof.**  According to Equations (13), (14), (16), and (18), we can obtain

$$\begin{array}{ccc} & & P_{i,k}^{\left(L\right)}=\left(I-K_{i,k}^{\left(L\right)}C_{i,k}\right)\left(P_{i,k|k-1}\right){\left(I-K_{i,k}^{\left(L\right)}C_{i,k}\right)}^{T}+K_{i,k}^{\left(L\right)}{\tilde{R}}_{i,k}^{\left(L\right)}{\left(K_{i,k}^{\left(L\right)}\right)}^{T},\\ & & K_{i,k}^{\left(L\right)}=\Gamma_{i,k}^{\left(L\right)}P_{i,k}^{dd,\left(L\right)}{\left(E_{k-1}\right)}^{T}{\left(C_{i,k}\right)}^{T}{\left({\overset{˘}{R}}_{i,k}^{\left(L\right)}\right)}^{-1}+P_{i,k|k-1}C_{i,k}^{T}{\left({\overset{˘}{R}}_{i,k}^{\left(L\right)}\right)}^{-1},\\ & & \Gamma_{i,k}^{\left(L\right)}=E_{k-1}-P_{i,k|k-1}C_{i,k}^{T}{\left({\overset{˘}{R}}_{i,k}^{\left(L\right)}\right)}^{-1}C_{i,k}E_{k-1}. \end{array}$$

Then we have

$$\begin{array}{ccc} & & \;P_{i,k}^{\left(L\right)}=K_{i,k}^{\left(L\right)}\left(C_{i,k}P_{i,k|k-1}C_{i,k}^{T}+{\tilde{R}}_{i,k}^{\left(L\right)}\right){\left(K_{i,k}^{\left(L\right)}\right)}^{T}-P_{i,k|k-1}{\left(C_{i,k}\right)}^{T}{\left(K_{i,k}^{\left(L\right)}\right)}^{T}+P_{i,k|k-1}\\ & & \;-K_{i,k}^{\left(L\right)}C_{i,k}P_{i,k|k-1}. \end{array}$$

Let

$$\begin{array}{ccc} & & \;\Sigma ={\overset{˘}{R}}_{i,k}^{\left(L\right)},\;L_{*}=K_{i,k}^{\left(L\right)},\;E=E_{k-1},\;{Ω}^{T}\Sigma^{-1}Ω={\left(P_{i,k}^{dd,\left(L\right)}\right)}^{-1},\;Ω=C_{i,k}E_{k-1},\;\Psi =C_{i,k}P_{i,k|k-1}. \end{array}$$

The relation $L_{*}Ω=E$ holds. Using Lemma 1, one can obtain that

(33) $$\begin{array}{ccc} & & \;P_{i,k}^{\left(L\right)}\leq \left(I-{\bar{L}}_{i,k}C_{i,k}\right)P_{i,k|k-1}{\left(I-{\bar{L}}_{i,k}C_{i,k}\right)}^{T}+{\bar{L}}_{i,k}{\tilde{R}}_{i,k}^{\left(L\right)}{\left({\bar{L}}_{i,k}\right)}^{T}, \end{array}$$

where ${\bar{L}}_{i,k}$ is a nonoptimal filter gain. According to Equations (10) and (25), we have

(34) $$\begin{array}{ccc} & & \;P_{i,k|k-1}\leq \frac{1}{a_{i,i}\left(T_{c}\right)}A_{k-1}P_{i,k-1}^{\left(L\right)}A_{k-1}^{T}+Q_{k-1}. \end{array}$$

Substituting Equation (34) into Equation (33) yields

(35) $$\begin{array}{ccc} & & \;P_{i,k}^{\left(L\right)}\leq \left(I-{\bar{L}}_{i,k}C_{i,k}\right)\frac{1}{\sqrt{a_{i,i}\left(T_{c}\right)}}A_{k-1}P_{i,k-1}^{\left(L\right)}{\left[\left(I-{\bar{L}}_{i,k}C_{i,k}\right)\frac{1}{\sqrt{a_{i,i}\left(T_{c}\right)}}A_{k-1}\right]}^{T}\\ & & \;+{\bar{L}}_{i,k}{\tilde{R}}_{i,k}^{\left(L\right)}{\left({\bar{L}}_{i,k}\right)}^{T}+\left(I-{\bar{L}}_{i,k}C_{i,k}\right)Q_{k-1}{\left(I-{\bar{L}}_{i,k}C_{i,k}\right)}^{T}. \end{array}$$

Construct a nonoptimal filter gain ${\bar{L}}_{i,k}$ satisfying the constraint ${\bar{L}}_{i,k}Ω=E$ as follows:

(36) $$\begin{array}{ccc} & & \;{\bar{L}}_{i,k}=G_{i,k}+{\bar{K}}_{k}-{\bar{K}}_{k}C_{i,k}G_{i,k}, \end{array}$$

where ${\bar{K}}_{k}$ is obtained by the classical Kalman filter given in Equation (29). Substituting Equation (36) into Equation (35), we have

(37) $$\begin{array}{ccc} P_{i,k}^{\left(L\right)} & & \leq \left(I-{\bar{K}}_{k}C_{i,k}\right){\bar{A}}_{i,k-1}P_{i,k-1}^{\left(L\right)}{\bar{A}}_{i,k-1}^{T}{\left(I-{\bar{K}}_{k}C_{i,k}\right)}^{T}+\left(I-{\bar{L}}_{i,k}C_{i,k}\right)Q_{k-1}{\left(I-{\bar{L}}_{i,k}C_{i,k}\right)}^{T}\\ & & \;+{\bar{L}}_{i,k}{\tilde{R}}_{i,k}^{\left(L\right)}{\left({\bar{L}}_{i,k}\right)}^{T}\\ & & \leq {\overset{˘}{A}}_{i,k-1}P_{i,k-1}^{\left(L\right)}{\left({\overset{˘}{A}}_{i,k-1}\right)}^{T}+{\bar{\Delta }}_{i,k}, \end{array}$$

where

$$\begin{array}{ccc} & & \;{\bar{\Delta }}_{i,k}=\left(I-{\bar{L}}_{i,k}C_{i,k}\right)Q_{k-1}{\left(I-{\bar{L}}_{i,k}C_{i,k}\right)}^{T}+{\bar{L}}_{i,k}{\tilde{R}}_{i,k}^{\left(L\right)}{\left({\bar{L}}_{i,k}\right)}^{T},\;{\overset{˘}{A}}_{i,k-1}=\left(I-{\bar{K}}_{k}C_{i,k}\right){\bar{A}}_{i,k-1}. \end{array}$$

By recursively applying Equation (37), we can obtain

(38) $$\begin{array}{ccc} & & \;P_{i,k}^{\left(L\right)}\leq \prod_{\xi =1}^{k-1}{\overset{˘}{A}}_{i,\xi }P_{i,1}^{\left(L\right)}{\left(\prod_{\xi =1}^{k-1}{\overset{˘}{A}}_{i,\xi }\right)}^{T}+{\bar{\Delta }}_{i,k}+\sum_{\xi =1}^{k-2}{\overset{˘}{A}}_{i,k-1}\ldots {\overset{˘}{A}}_{i,k-\xi }{\bar{\Delta }}_{i,k-\xi }{\left({\overset{˘}{A}}_{i,k-1}\ldots {\overset{˘}{A}}_{i,k-\xi }\right)}^{T}. \end{array}$$

Taking the 2-norm of both sides of Equation (38), we can obtain

(39) $$\begin{array}{ccc} & & \;∥P_{i,k}^{\left(L\right)}∥\leq {∥\prod_{\xi =1}^{k-1}{\overset{˘}{A}}_{i,\xi }∥}^{2}∥P_{i,1}^{\left(L\right)}∥+∥{\bar{\Delta }}_{i,k}∥+\sum_{\xi =1}^{k-2}{∥{\overset{˘}{A}}_{i,k-1}\ldots {\overset{˘}{A}}_{i,k-\xi }∥}^{2}∥{\bar{\Delta }}_{i,k-\xi }∥. \end{array}$$

According to Proposition 1 of [39], if conditions (i)–(iv) hold, then ${\bar{L}}_{i,k}$ is bounded for all k≥1. Thus, there exists a positive constant $\bar{\delta }$ such that $∥{\bar{\Delta }}_{i,k}∥\leq \bar{\delta }$ holds for all k≥1. According to [39] and Corollary 1 of [41], if the matrix sequence pair $\left[{\bar{A}}_{i,k},C_{i,k}\right]$ is uniformly completely observable, and the matrix sequence pair $\left[{\bar{A}}_{i,k},I\right]$ is uniformly completely controllable (trivially satisfied because the input matrix is the identity matrix), then the error system of the classical Kalman filter Equations (28)–(32) is exponentially stable, that is,

(40) $$\begin{array}{ccc} & & \;∥{\overset{˘}{A}}_{i,k-1}\ldots {\overset{˘}{A}}_{i,1}∥\leq \alpha e^{-\beta \left(k-1\right)}. \end{array}$$

Substituting Equation (40) into Equation (39), we can obtain

(41) $$\begin{array}{ccc} & & \;∥P_{i,k}^{\left(L\right)}∥\leq \alpha^{2}{\bar{p}}_{1}e^{-2\beta \left(k-1\right)}+\alpha^{2}\bar{\delta }\frac{e^{-2\beta }\left(1-e^{-2\beta \left(k-2\right)}\right)}{1-e^{-2\beta }}+\bar{\delta }, \end{array}$$

where $∥P_{i,1}^{\left(L\right)}∥\leq {\bar{p}}_{1}$. The boundedness of $∥P_{i,1}^{\left(L\right)}∥$ follows from conditions (ii) and (v). Equation (41) then implies that there exists a positive constant $\bar{p}$ such that $P_{i,k}^{\left(L\right)}\leq \bar{p}I$. This completes the proof. □

**Theorem** **2.** *Consider the distributed filter given in Algorithm 1. Let $e_{i,k}=x_{k}-{\hat{x}}_{i,k}$ denote the estimation error of sensor node i. For any i∈V, the unknown input $d_{k-1}$ does not influence the estimation error $e_{i,k}$. In other words, $e_{i,k}$ does not contain $d_{k-1}$.*

**Proof.**  Let $e_{i,k}^{\left(l\right)}=x_{k}-{\hat{x}}_{i,k}^{\left(l\right)}$, l∈{1,…,L}. Firstly, we will prove that $d_{k-1}$ does not influence $e_{i,k}^{\left(1\right)},\ldots ,e_{i,k}^{\left(L\right)}$ and ${\hat{\lambda }}_{i,k}^{\left(1\right)},\ldots ,{\hat{\lambda }}_{i,k}^{\left(L\right)}$, which can be proved by induction. From Equation (17), we can obtain

(42) $$\begin{array}{ccc} & & \;e_{i,k}^{\left(l\right)}=\left(I-K_{i,k}^{\left(l\right)}C_{i,k}\right)A_{k-1}e_{i,k-1}+\left(I-K_{i,k}^{\left(l\right)}C_{i,k}\right)\omega_{k-1}-K_{i,k}^{\left(l\right)}\varepsilon_{i,k}, \end{array}$$

where l∈{1,…,L} and

$$\begin{array}{ccc} & & \;K_{i,k}^{\left(l\right)}=\Gamma_{i,k}^{\left(l\right)}P_{i,k}^{dd,\left(l\right)}{\left(E_{k-1}\right)}^{T}{\left(C_{i,k}\right)}^{T}{\left({\overset{˘}{R}}_{i,k}^{\left(l\right)}\right)}^{-1}+P_{i,k|k-1}C_{i,k}^{T}{\left({\overset{˘}{R}}_{i,k}^{\left(l\right)}\right)}^{-1},\\ & & \;\Gamma_{i,k}^{\left(l\right)}=E_{k-1}-P_{i,k|k-1}C_{i,k}^{T}{\left({\overset{˘}{R}}_{i,k}^{\left(l\right)}\right)}^{-1}C_{i,k}E_{k-1}. \end{array}$$

For l=1, we can conclude from Equation (42) and Equation (12) that both $e_{i,k}^{\left(1\right)}$ and ${\hat{\lambda }}_{i,k}^{\left(1\right)}$ do not contain $d_{k-1}$. Assume for $\hat{l}\in \left\{2,\ldots ,L-1\right\}$, $e_{i,k}^{\left(\hat{l}\right)}$ and ${\hat{\lambda }}_{i,k}^{\left(\hat{l}\right)}$ do not contain $d_{k-1}$. Based on Equations (19) and (20), we obtain

$$\begin{array}{ccc} & & \;\Delta_{i,k}^{\left(\hat{l}\right)}=\left(C_{i,k}e_{i,k}^{\left(\hat{l}\right)}+\varepsilon_{i,k}\right){\left(C_{i,k}e_{i,k}^{\left(\hat{l}\right)}+\varepsilon_{i,k}\right)}^{T}+C_{i,k}P_{i,k}^{\left(\hat{l}\right)}C_{i,k}^{T},\\ & & \;{\hat{\lambda }}_{i,k}^{\left(\hat{l}+1\right)}=\frac{\nu_{i,k}+n_{y}}{\mathrm{tr}\left(\Delta_{i,k}^{\left(\hat{l}\right)}R_{i,k}^{-1}\right)+\nu_{i,k}}. \end{array}$$

It follows that ${\hat{\lambda }}_{i,k}^{\left(\hat{l}+1\right)}$ depends only on $\nu_{i,k}$, $n_{y}$, $R_{i,k}$ and $\Delta_{i,k}^{\left(\hat{l}\right)}$. Thus, the filter gain $K_{i,k}^{\left(\hat{l}+1\right)}$ is not affected by $d_{k-1}$. From Equation (42), we can obtain that $e_{i,k}^{\left(\hat{l}+1\right)}$ does not contain $d_{k-1}$. By induction, it can be concluded that both $e_{i,k}^{\left(1\right)},\ldots ,e_{i,k}^{\left(L\right)}$ and ${\hat{\lambda }}_{i,k}^{\left(1\right)},\ldots ,{\hat{\lambda }}_{i,k}^{\left(L\right)}$ do not contain $d_{k-1}$. From Equations (21)–(25), we obtain

$$\begin{array}{ccc} & & \;e_{i,k}={\left[\sum_{j\in V}a_{i,j}\left(T_{c}\right){\left(P_{j,k}\right)}^{-1}\right]}^{-1}\sum_{j\in V}a_{i,j}\left(T_{c}\right){\left(P_{j,k}\right)}^{-1}e_{j,k}^{\left(L\right)}. \end{array}$$

Therefore, $d_{k-1}$ does not influence $e_{i,k}$. This completes the proof. □

## 5. Numerical Examples

**Figure 2:**
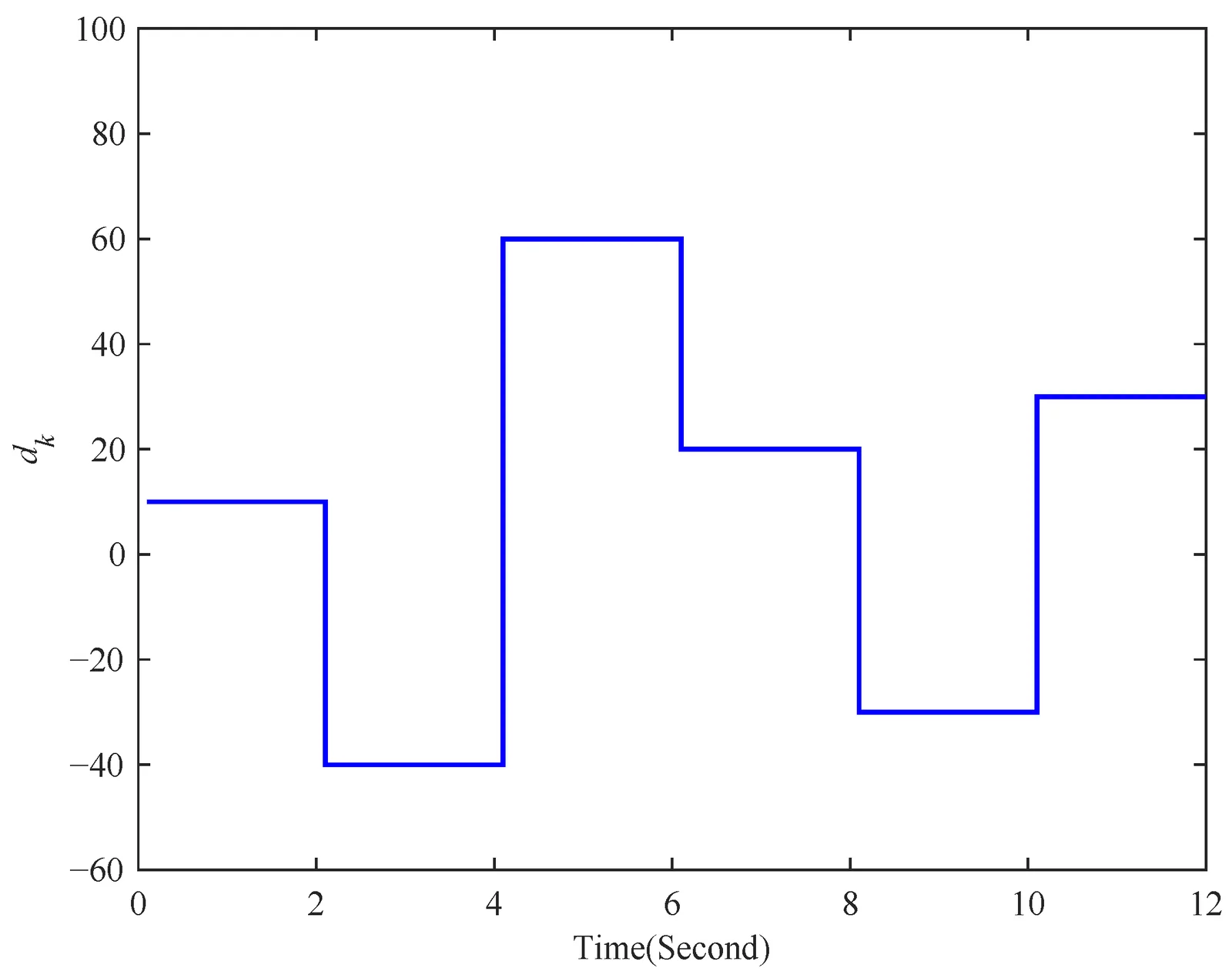
The unknown input signal.

In this section, a target tracking example is employed to verify the effectiveness of the proposed distributed filter. The motion model of the target is given by

(43) $$\begin{array}{ccc} & & \;x_{k+1}=Ax_{k}+Ed_{k}+\Gamma \omega_{k}, \end{array}$$

where $x_{k}={\left[p_{x,k},v_{x,k},p_{y,k},v_{y,k}\right]}^{T}$ is the state vector, and $\left(p_{x,k},p_{y,k}\right)$ and $\left(v_{x,k},v_{y,k}\right)$ denote the Cartesian coordinates and corresponding velocities, respectively. The process noise $\omega_{k}$ is zero-mean Gaussian noise with covariance $Q_{k-1}=0.1I$. Matrices *A*, *E*, and Γ are given by

$$\begin{array}{ccc} & & \;A=\left[\begin{array}{cccc} 1 & T & 0 & 0\\ 0 & 1 & 0 & 0\\ 0 & 0 & 1 & T\\ 0 & 0 & 0 & 1 \end{array}\right],\;E=\left[\begin{array}{c} T\\ 1\\ T\\ 1 \end{array}\right],\;\Gamma =\left[\begin{array}{cc} \frac{1}{2}T^{2} & 0\\ T & 0\\ 0 & \frac{1}{2}T^{2}\\ 0 & T \end{array}\right], \end{array}$$

where T=0.1 s is the sampling period. The unknown input signal $d_{k}$ is shown in Figure 2.

**Figure 3:**
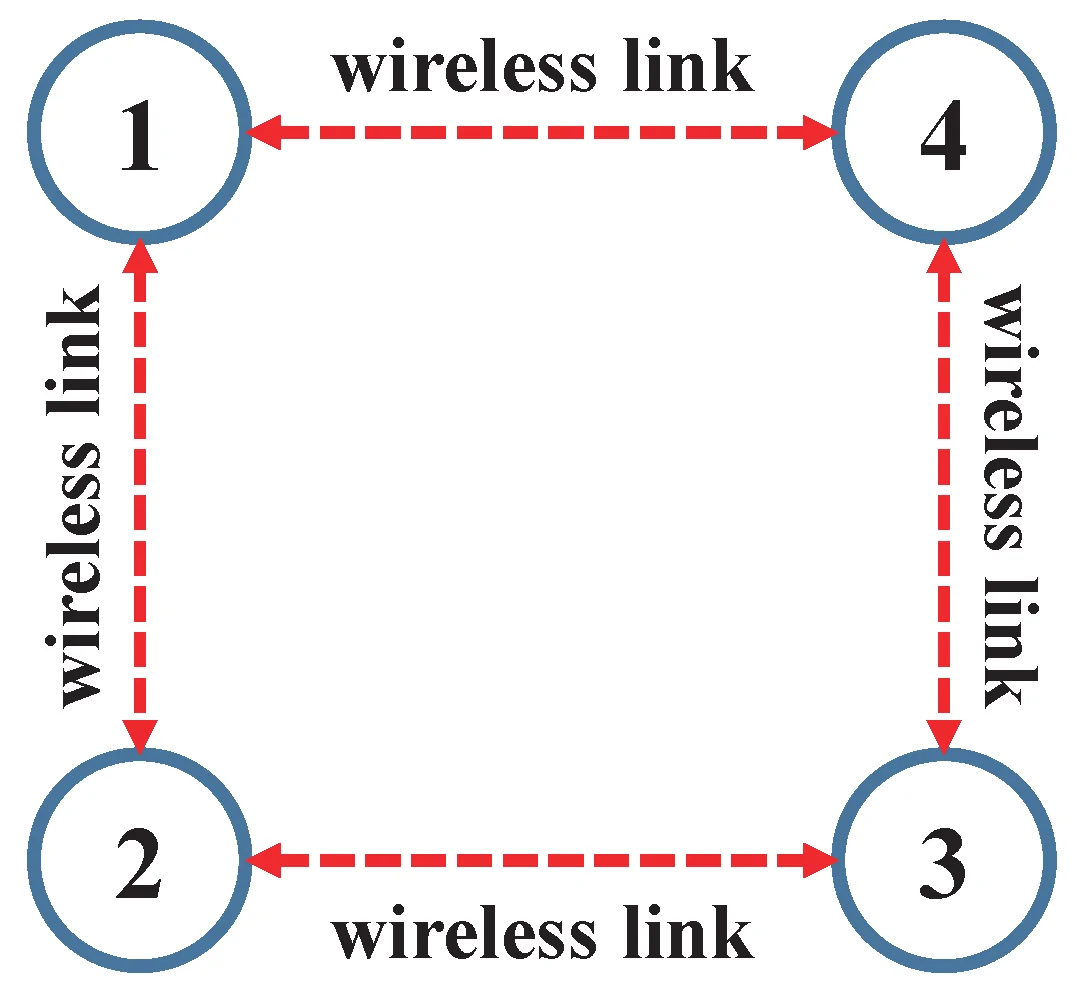
Communication topology of the sensor network.

The target is monitored by a sensor network composed of four nodes. The communication topology is depicted in Figure 3. The measurement model is $y_{i,k}=C_{i}x_{k}+\varepsilon_{i,k}$, i=1,2,3,4, where

$$\begin{array}{ccc} & & \;C_{i}=\left[\begin{array}{cccc} 1 & 0 & 0 & 0\\ 0 & 0 & 1 & 0 \end{array}\right]. \end{array}$$

The measurement noise $\varepsilon_{i,k}$ is considered to exhibit heavy-tailed characteristics and is generated according to the following Gaussian mixture model:

$$\begin{array}{ccc} & & \;\varepsilon_{i,k}∼\left\{\begin{array}{cc} N\left(0,R_{n}\right), & \mathrm{with}\;\mathrm{probability}\;0.9,\\ N\left(0,100R_{n}\right), & \mathrm{with}\;\mathrm{probability}\;0.1, \end{array}\right. \end{array}$$

where $R_{n}$ is selected as 0.01I.

The estimation accuracy of five algorithms is compared: (1) Algorithm 1; (2) a centralized variational Bayesian (VB) filter based on [29]; (3) a distributed Gaussian unknown-input filter; (4) the distributed filter in [5]; (5) a distributed extension of the VB filter based on [29]. Algorithm 1 adopts the measurement-noise model $p\left(\varepsilon_{i,k}\right)=\mathrm{St}\left(\varepsilon_{i,k};0,0.01I_{2},3\right)$. Its parameters are L=6 and $\alpha_{i,k}^{\left(0\right)}=\beta_{i,k}^{\left(0\right)}=1$. All distributed methods use the same network and consensus weights, with $T_{c}=2$. The centralized VB filter uses the stacked measurement model $y_{k}=Cx_{k}+\varepsilon_{k}$, where $y_{k}=\mathrm{col}{\left\{y_{i,k}\right\}}_{i=1}^{4}$, $C=\mathrm{col}{\left\{C_{i}\right\}}_{i=1}^{4}$, and $\varepsilon_{k}=\mathrm{col}{\left\{\varepsilon_{i,k}\right\}}_{i=1}^{4}$. Its noise model is $p\left(\varepsilon_{k}\right)=\mathrm{St}\left(\varepsilon_{k};0,0.01I_{8},3\right)$. The distributed VB extension performs single-sensor VB updates locally. It then fuses the state information vectors and matrices through consensus. The two Gaussian filters assume $p\left(\varepsilon_{i,k}\right)=N\left(\varepsilon_{i,k};0,0.01I_{2}\right)$. The filter in [5] uses local Kalman updates and posterior information consensus. All five methods use the same trajectory and measurement realizations within each Monte Carlo trial.

The position and velocity RMSEs, ${\mathrm{RMSE}}_{p,k}$ and ${\mathrm{RMSE}}_{v,k}$, are used to evaluate estimation accuracy. The position RMSE is defined as

$${\mathrm{RMSE}}_{p,k}={\left[\frac{1}{N\Theta }\sum_{i=1}^{N}\sum_{\vartheta =1}^{\Theta }\left({\left(p_{x,k}^{\vartheta }-{\hat{p}}_{x,i,k}^{\vartheta }\right)}^{2}+{\left(p_{y,k}^{\vartheta }-{\hat{p}}_{y,i,k}^{\vartheta }\right)}^{2}\right)\right]}^{1/2},$$

where Θ=100 is the number of Monte Carlo trials. For this metric, N=1 for the centralized estimate and N=4 for the distributed estimates. The superscript ϑ denotes the trial index. The variables $p_{x,k}^{\vartheta }$ and $p_{y,k}^{\vartheta }$ are the true position components. Their hatted counterparts are the estimates produced by node *i*. The velocity RMSE is defined analogously.

**Figure 4:**
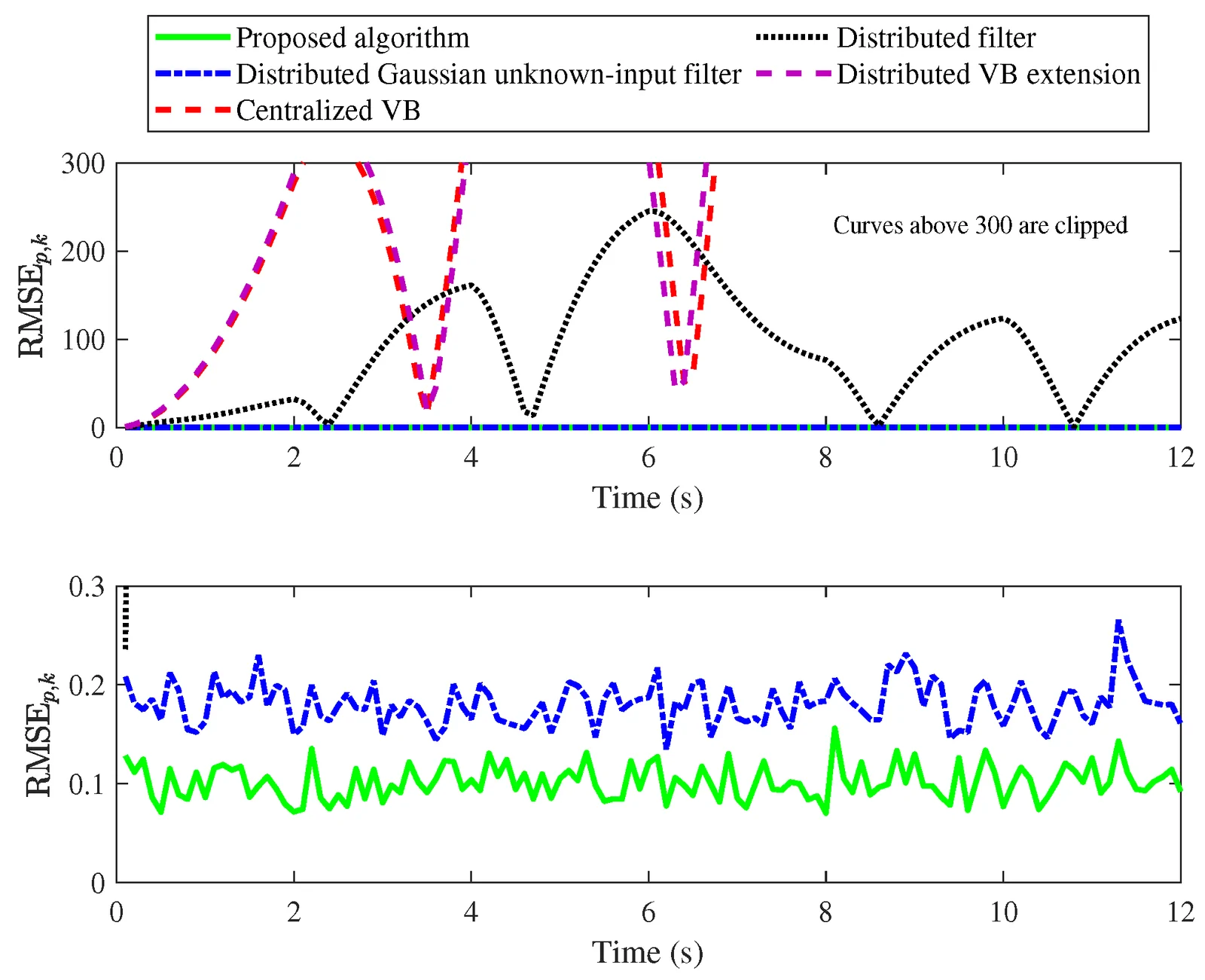
${\mathrm{RMSE}}_{p,k}$ of different algorithms: overview (**top**) and zoomed-in view (**bottom**). The centralized VB filter and its distributed extension are based on [29]; the distributed filter follows the method in [5].

**Figure 5:**
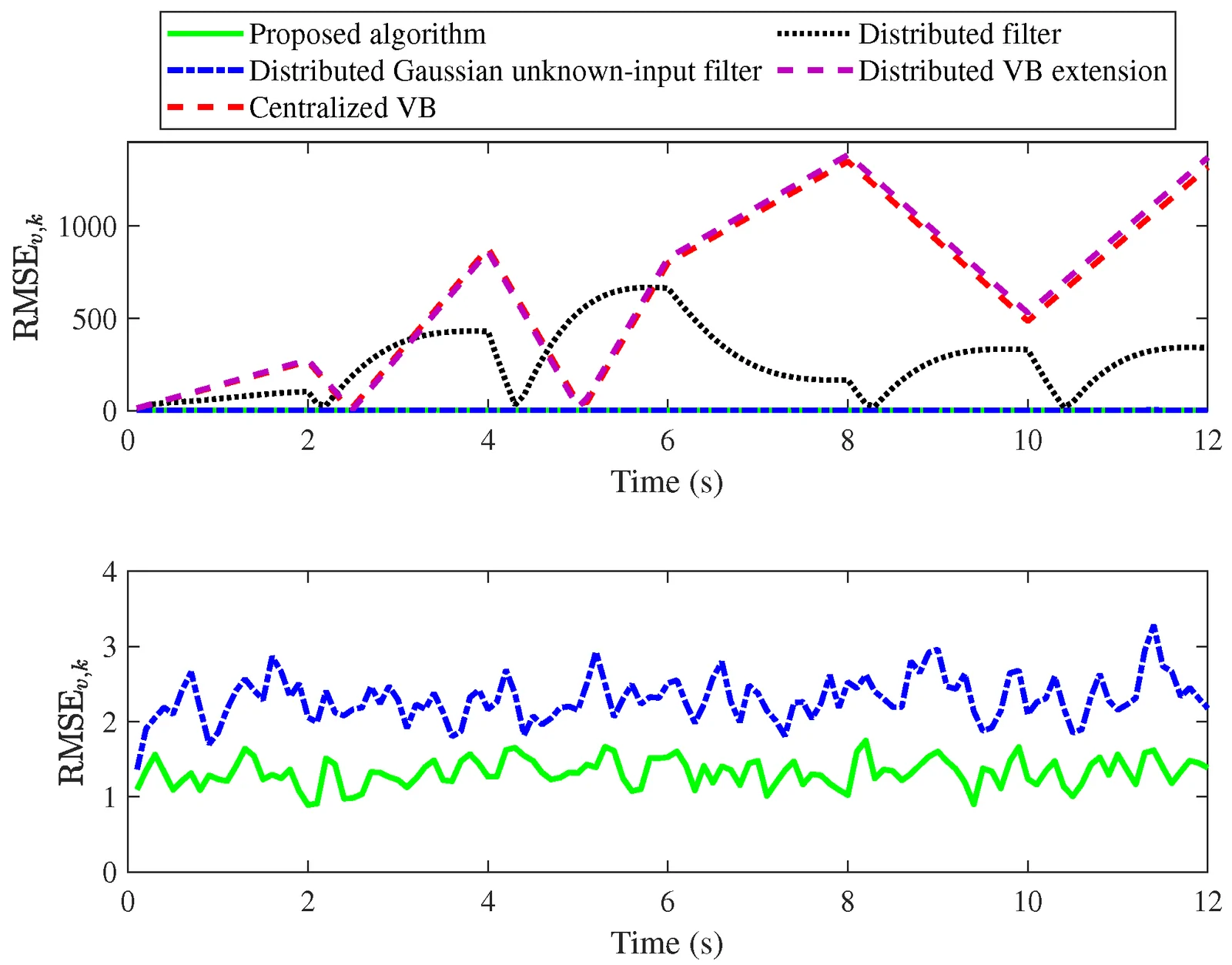
${\mathrm{RMSE}}_{v,k}$ of different algorithms: overview (**top**) and zoomed-in view (**bottom**). The centralized VB filter and its distributed extension are based on [29]; the distributed filter follows the method in [5].

The position and velocity RMSEs are presented in Figure 4 and Figure 5, respectively. Algorithm 1 achieves lower RMSEs than the four benchmarks under the investigated conditions. The enlarged views show that the distributed Gaussian unknown-input filter also maintains relatively low errors. Both methods employ non-informative priors for the unknown inputs. They therefore avoid imposing a specific input evolution model. The proposed algorithm further reduces the estimation errors through its Student’s *t* measurement-noise model. This comparison supports the benefit of robust noise modeling under the considered outlier contamination. The distributed filter in [5] produces substantially larger position and velocity errors. This behavior is consistent with its lack of explicit unknown-input compensation. Its Gaussian measurement-noise assumption also limits its robustness under the considered noise conditions. Both VB implementations based on [29] exhibit large errors. The distributed extension shows similar error behavior to the centralized implementation and provides no clear accuracy improvement. These implementations propagate Gaussian unknown-input priors. The predicted input means are taken from the preceding input estimates. Such priors may introduce lag when the unknown input changes abruptly. This may contribute to the observed errors.

**Figure 6:**
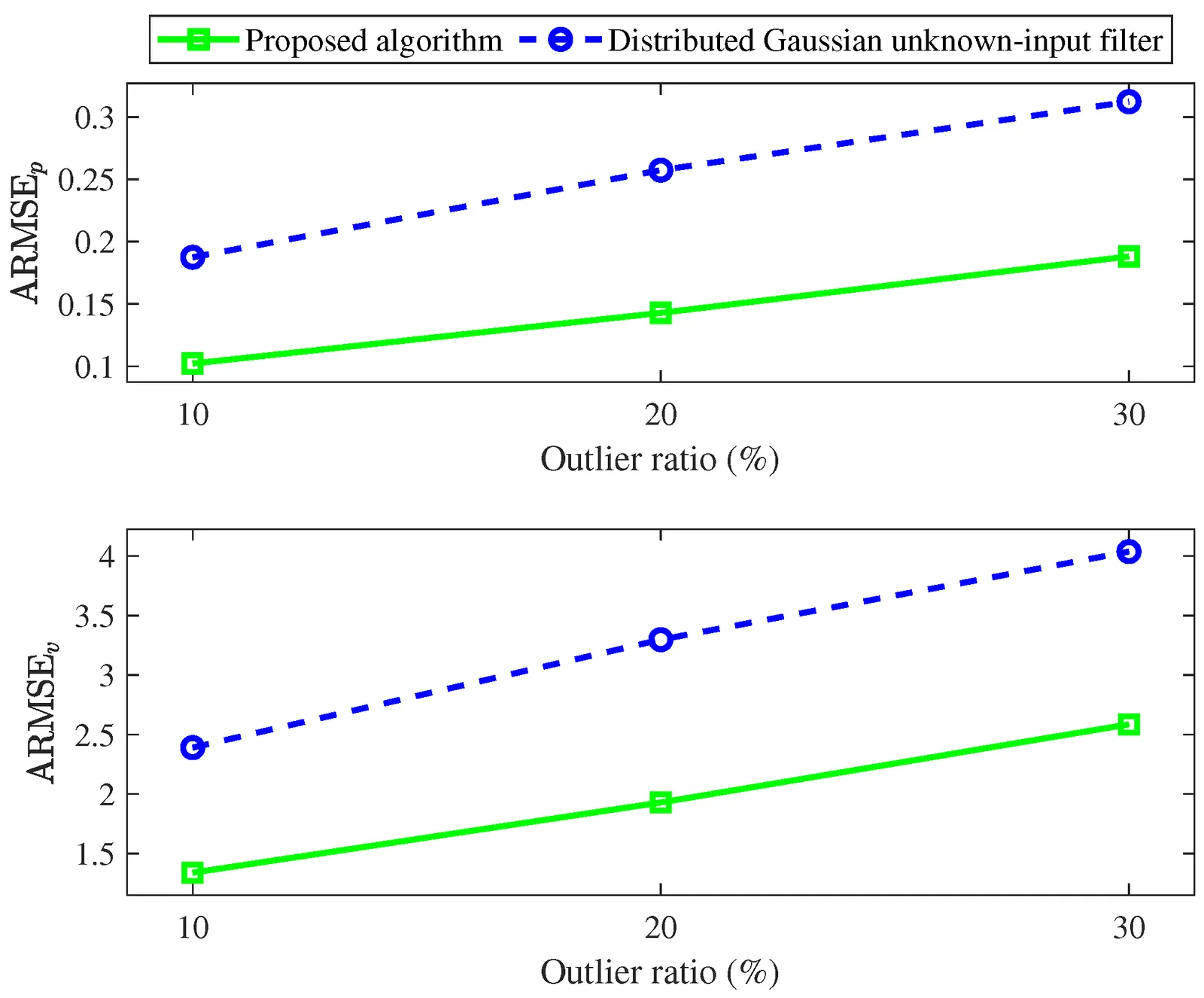
Position and velocity ARMSEs under different outlier ratios.

To further evaluate the robustness against outliers, the proposed algorithm is compared with the distributed Gaussian unknown-input filter under three outlier ratios: 10%, 20%, and 30%. Only the outlier ratio is varied. The average root mean square error (ARMSE) is defined as ${\mathrm{ARMSE}}_{s}=\frac{1}{K}\sum_{k=1}^{K}{\mathrm{RMSE}}_{s,k}$, where *K* is the number of time steps and s∈{p,v} denotes position or velocity. Figure 6 shows the position and velocity ARMSEs of the two algorithms. As the outlier ratio increases, the ARMSEs of both algorithms increase. However, the proposed algorithm consistently achieves lower ARMSEs. At an outlier ratio of 30%, its position and velocity ARMSEs are 0.18 and 2.5, compared with 0.31 and 4.0 for the Gaussian filter. These results demonstrate the improved robustness of the proposed algorithm against measurement outliers.

To assess the effect of unknown-input amplitude on Algorithm 1, two cases are considered: γ=1 and γ=2. The motion model is $x_{k+1}=Ax_{k}+\gamma Ed_{k}+\Gamma \omega_{k}$. Here, *A*, *E*, Γ, and $\omega_{k}$ are the same as in Equation (43). The factor γ scales the amplitude of the step signal shown in Figure 2. Algorithm 1 is evaluated over 100 Monte Carlo trials. Within each trial, the two cases share the same process noise, measurement noise, outlier locations, and initial conditions. All filter parameters are identical.

**Figure 7:**
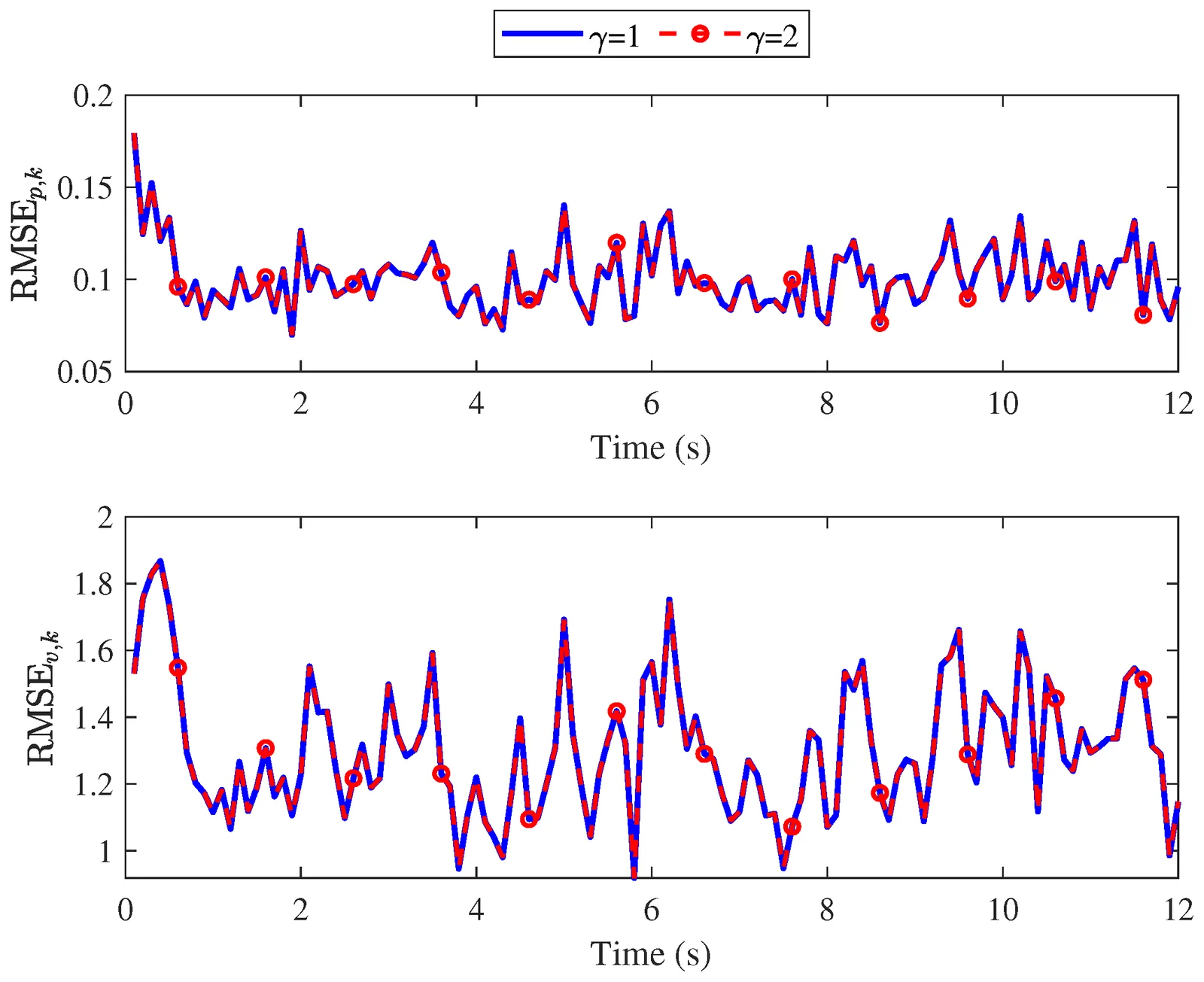
${\mathrm{RMSE}}_{p,k}$ and ${\mathrm{RMSE}}_{v,k}$ for the original (γ=1) and doubled (γ=2) unknown-input amplitudes.

Figure 7 shows that the RMSE curves of the two cases overlap throughout the simulation. Doubling the unknown-input amplitude produces no visible increase in either RMSE. This agreement also holds around the input changes near 2, 6, and 10 s.

**Remark** **3.** *In the simulations, the number of fixed-point iterations is set to L=6. Using scalar multiplications and divisions to measure computational cost, the additional per-node computational complexity of the proposed filter compared with the standard Kalman consensus filter is $O(L\left(n_{x}^{2}n_{y}+n_{x}n_{y}^{2}+n_{y}^{3}\right))$, where $n_{x}$ and $n_{y}$ denote the state and measurement dimensions, respectively. Thus, the additional computational cost increases linearly with L. Since the VB iterations are performed locally, the communication load is independent of L. Monte Carlo results show that increasing L from 6 to 10 reduces the ARMSEs of position and velocity by only 0.70% and 0.22%, respectively. For practical applications, a small fixed L can be selected through offline Monte Carlo tests to meet the required estimation accuracy. This provides a balance between estimation accuracy and computational cost for resource-constrained sensor nodes.*

**Figure 8:**
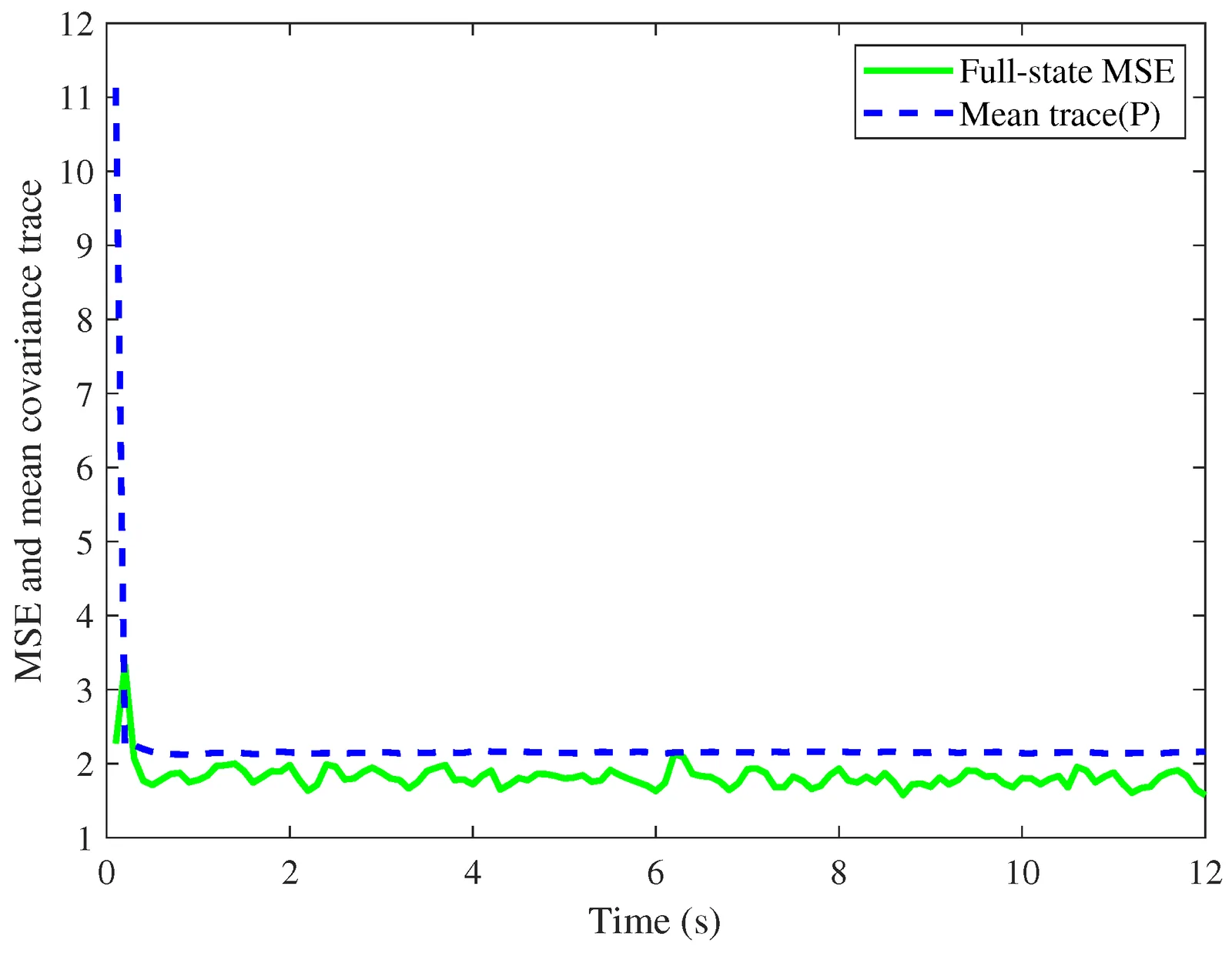
Full-state MSE and averaged covariance trace over 3000 Monte Carlo trials and four sensor nodes.

To examine the relationship between estimation errors and the covariance reported by Algorithm 1, we perform 3000 Monte Carlo trials with L=6. The full-state MSE is defined as ${\mathrm{MSE}}_{k}=\frac{1}{N\Theta }\sum_{i=1}^{N}\sum_{\vartheta =1}^{\Theta }{∥x_{k}^{\vartheta }-{\hat{x}}_{i,k}^{\vartheta }∥}_{2}^{2}$. The averaged covariance trace is defined as ${\bar{\mathrm{tr}(P)}}_{k}=\frac{1}{N\Theta }\sum_{i=1}^{N}\sum_{\vartheta =1}^{\Theta }\mathrm{tr}\left(P_{i,k}^{\vartheta }\right)$. Here, N=4 and Θ=3000 for this experiment. Both ${\hat{x}}_{i,k}^{\vartheta }$ and $P_{i,k}^{\vartheta }$ are obtained after consensus. As shown in Figure 8, ${\mathrm{MSE}}_{k}$ does not exceed ${\bar{\mathrm{tr}(P)}}_{k}$ at 119 of the 120 sampling instants (99.17%). Their time-averaged values are 1.8255 and 2.2270, respectively. At the remaining sampling instant, the MSE exceeds the averaged covariance trace by 1.1209. These results show that the averaged covariance trace is larger than the MSE at most sampling instants under the tested conditions.

## 6. Conclusions

This paper investigated distributed filtering problems for linear systems with unknown inputs and heavy-tailed measurement noises. Firstly, as a basis for designing the distributed filter, a local filter was designed for a single-sensor system based on the variational Bayesian method. Secondly, a distributed filter was proposed for sensor networks by combining the local filter with the consensus on information method. Thirdly, a sufficient condition was derived for the boundedness of the estimation error covariance. Finally, the effectiveness of the distributed filter was verified through simulations. The simulation results demonstrate that the proposed distributed filter achieves better estimation performance than the compared methods under the considered scenarios with unknown inputs and heavy-tailed measurement noises. Future work will focus on extending the proposed framework to other heavy-tailed noise models, such as the Laplace distribution and Gaussian mixture models.

## Figures and Tables

**Table 1:** Summary of existing state estimation methods for systems with unknown inputs.

References	Fusion Structure	Noise	Design Methods	Stability Analysis
[16]	Distributed	Gaussian noise	Unbiased minimum variance criterion	✔
[17]	Distributed	Gaussian noise	Unbiased minimum variance criterion	✔
[18]	Distributed	Gaussian noise	Minimization of an upper bound on the error covariance	✔
[19]	Distributed	Gaussian noise	Bayesian methods and non-informative prior distribution	✔
[29]	Single sensor/centralized	Heavy-tailed noise	Variational Bayesian methods	×
[30]	Single sensor/centralized	Heavy-tailed noise	Statistical linearization and maximum correntropy criterion	×

## Data Availability

The datasets presented in this article are not readily available because they consist of numerical simulation data generated specifically for this study and have not been deposited in a public repository. Requests to access the datasets should be directed to the corresponding author.

## Appendix A. Derivation of the Local Filter

The design procedure of the local filter proposed in Section 3 is given in this appendix. Using the law of total probability together with Equations (1) and (5), we obtain

(A1) $$\begin{array}{ccc} & & \;p\left(x_{k}|d_{k-1},y_{i,1:k-1}\right)\\ & & \;=\int p\left(x_{k}|x_{k-1},d_{k-1}\right)p\left(x_{k-1}|d_{k-1},y_{i,1:k-1}\right)dx_{k-1}\\ & & \;=\int N\left(x_{k};A_{k-1}x_{k-1}+E_{k-1}d_{k-1},Q_{k-1}\right)N\left(x_{k-1};{\hat{x}}_{i,k-1},P_{i,k-1}\right)dx_{k-1}\\ & & \;=N\left(x_{k};{\hat{x}}_{i,k|k-1}+E_{k-1}d_{k-1},P_{i,k|k-1}\right), \end{array}$$

where ${\hat{x}}_{i,k|k-1}$ and $P_{i,k|k-1}$ are calculated by Equations (9) and (10), respectively.

To simultaneously estimate the state $x_{k}$ and the unknown input $d_{k-1}$ of a hierarchical Gaussian state-space model, we need to compute the joint posterior PDF $p(x_{k},\lambda_{i,k},d_{k-1}|y_{i,1:k})$. For the hierarchical Gaussian state-space model, there is no analytical solution for this posterior PDF. Thus, to obtain an approximate solution, the VB approach is used to find a factorized approximation to the joint posterior PDF, i.e.,

$$\begin{array}{ccc} & & \;p\left(x_{k},d_{k-1},\lambda_{i,k}|y_{i,1:k}\right)\approx q\left(x_{k},d_{k-1}\right)q\left(\lambda_{i,k}\right), \end{array}$$

where q(·) is the approximate posterior PDF. According to the VB approach, these approximate posterior PDFs can be obtained by minimizing the KLD between the approximate posterior PDF $q\left(x_{k},d_{k-1}\right)q\left(\lambda_{i,k}\right)$ and the true posterior PDF $p\left(x_{k},d_{k-1},\lambda_{i,k}|y_{i,1:k}\right)$, i.e.,

(A2) $$\begin{array}{ccc} & & \;\left\{q\left(x_{k},d_{k-1}\right)q\left(\lambda_{i,k}\right)\right\}=\underset{\hat{q}\left(x_{k},d_{k-1}\right)\hat{q}\left(\lambda_{i,k}\right)}{\arg\min\mathrm{KLD}}\left\{\hat{q}\left(x_{k},d_{k-1}\right)\hat{q}\left(\lambda_{i,k}\right)\left|\right|p\left(x_{k},d_{k-1},\lambda_{i,k}|y_{i,1:k}\right)\right\}. \end{array}$$

The optimal solution to Equation (A2) satisfies the following equation:

(A3) $$\begin{array}{ccc} & & \;\ln q\left(\phi \right)=E_{\Theta^{-\phi }}\left[\ln p\left(\Theta ,y_{i,1:k}\right)\right]+c_{\phi },\;\Theta =\left\{\left\{x_{k},d_{k-1}\right\},\lambda_{i,k}\right\}, \end{array}$$

where ϕ denotes a variable or a group of variables in Θ, and $\Theta^{-\phi }$ denotes the remaining variables. Here, $E_{\Theta^{-\phi }}\left[\cdot \right]$ denotes the expectation operator with respect to the variational posterior PDF of $\Theta^{-\phi }$. For example, $E_{\lambda_{i,k}}\left[\cdot \right]$ denotes the expectation with respect to $q(\lambda_{i,k})$. Moreover, $c_{\phi }$ is a constant independent of ϕ. According to Equations (2), (7), (8) and (A1), we can obtain

(A4) $$\begin{array}{ccc} & & \;p\left(y_{i,k},x_{k},d_{k-1},\lambda_{i,k},y_{i,1:k-1}\right)\\ & & \;=p\left(y_{i,k}|x_{k},\lambda_{i,k}\right)p\left(x_{k}|d_{k-1},y_{i,1:k-1}\right)p\left(d_{k-1}|y_{i,1:k-1}\right)p\left(\lambda_{i,k}|y_{i,1:k-1}\right)p\left(y_{i,1:k-1}\right)\\ & & \;\propto N\left(y_{i,k};C_{i,k}x_{k},R_{i,k}/\lambda_{i,k}\right)G\left(\lambda_{i,k};\frac{\nu_{i,k}}{2},\frac{\nu_{i,k}}{2}\right)N\left(x_{k};{\hat{x}}_{i,k|k-1}+E_{k-1}d_{k-1},P_{i,k|k-1}\right)\\ & & \;\times p\left(y_{i,1:k-1}\right). \end{array}$$

Substituting Equation (A4) into Equation (A3) and setting $\phi =\lambda_{i,k}$, we obtain

(A5) $$\begin{array}{ccc} & & \;\ln q\left(\lambda_{i,k}\right)\\ & & \;=E_{x_{k},d_{k-1}}\left[\ln N\left(y_{i,k};C_{i,k}x_{k},R_{i,k}/\lambda_{i,k}\right)G\left(\lambda_{i,k};\frac{\nu_{i,k}}{2},\frac{\nu_{i,k}}{2}\right)\right]+c_{\lambda_{i,k}}\\ & & \;=\left(\frac{\nu_{i,k}+n_{y}}{2}-1\right)\ln\left(\lambda_{i,k}\right)-\frac{1}{2}\left[\mathrm{tr}\left(\Delta_{i,k}R_{i,k}^{-1}\right)+\nu_{i,k}\right]\lambda_{i,k}, \end{array}$$

where

(A6) $$\begin{array}{ccc} & & \;\Delta_{i,k}=E_{x_{k}}\left[\left(y_{i,k}-C_{i,k}x_{k}\right){\left(y_{i,k}-C_{i,k}x_{k}\right)}^{T}\right]. \end{array}$$

Then we obtain

$$\begin{array}{ccc} & & \;q\left(\lambda_{i,k}\right)=G\left(\lambda_{i,k};\alpha_{i,k},\beta_{i,k}\right), \end{array}$$

where

(A7) $$\begin{array}{ccc} & & \;\alpha_{i,k}=\frac{\nu_{i,k}+n_{y}}{2},\;\beta_{i,k}=\frac{1}{2}\left[\mathrm{tr}\left(\Delta_{i,k}R_{i,k}^{-1}\right)+\nu_{i,k}\right]. \end{array}$$

Setting $\phi =\left\{x_{k},d_{k-1}\right\}$, we obtain

(A8) $$\begin{array}{ccc} & & \;\ln q\left(x_{k},d_{k-1}\right)\\ & & \;=E_{\lambda_{i,k}}\left[\ln N\left(y_{i,k};C_{i,k}x_{k},R_{i,k}/\lambda_{i,k}\right)N\left(x_{k};{\hat{x}}_{i,k|k-1}+E_{k-1}d_{k-1},P_{i,k|k-1}\right)\right]+c_{x_{k},d_{k-1}}\\ & & \;=-\frac{1}{2}{\left(x_{k}-{\hat{x}}_{i,k|k-1}-E_{k-1}d_{k-1}\right)}^{T}{\left(P_{i,k|k-1}\right)}^{-1}\left(x_{k}-{\hat{x}}_{i,k|k-1}-E_{k-1}d_{k-1}\right)\\ & & \;-\frac{1}{2}{\left(y_{i,k}-C_{i,k}x_{k}\right)}^{T}{\tilde{R}}_{i,k}^{-1}\left(y_{i,k}-C_{i,k}x_{k}\right)+c_{x_{k},d_{k-1}}, \end{array}$$

where

(A9) $$\begin{array}{ccc} & & \;{\tilde{R}}_{i,k}=R_{i,k}/{\hat{\lambda }}_{i,k},\;{\hat{\lambda }}_{i,k}=\frac{\alpha_{i,k}}{\beta_{i,k}}. \end{array}$$

Here, ${\hat{\lambda }}_{i,k}$ denotes the mean of the auxiliary random variable $\Lambda_{i,k}$ under the variational posterior PDF $q(\lambda_{i,k})$. Using matrix transformation and the matrix inverse lemma, if ${\left(C_{i,k}E_{k-1}\right)}^{T}{\left({\tilde{R}}_{i,k}+C_{i,k}P_{i,k|k-1}C_{i,k}^{T}\right)}^{-1}C_{i,k}E_{k-1}$ is invertible, then Equation (A8) can be further written as

(A10) $$\begin{array}{ccc} & & \;q\left(x_{k},d_{k-1}\right)=N\left(\left[\begin{array}{c} x_{k}\\ d_{k-1} \end{array}\right];\mu_{i,k},\Sigma_{i,k}\right), \end{array}$$

where

(A11) $$\begin{array}{ccc} & & \;\mu_{i,k}=\left[\begin{array}{c} {\hat{x}}_{i,k}\\ {\hat{d}}_{i,k-1} \end{array}\right],\;\Sigma_{i,k}=\left[\begin{array}{cc} P_{i,k} & P_{i,k}^{xd}\\ {\left(P_{i,k}^{xd}\right)}^{T} & P_{i,k}^{dd} \end{array}\right]. \end{array}$$

In Equation (A11), ${\hat{x}}_{i,k}$ and ${\hat{d}}_{i,k-1}$ are calculated by

(A12) $$\begin{array}{ccc} & & \;{\hat{x}}_{i,k}={\hat{x}}_{i,k|k-1}+P_{i,k}^{xd}{\left(P_{i,k}^{dd}\right)}^{-1}{\hat{d}}_{i,k-1}+P_{i,k|k-1}C_{i,k}^{T}{\overset{˘}{R}}_{i,k}^{-1}\left(y_{i,k}-C_{i,k}{\hat{x}}_{i,k|k-1}\right), \end{array}$$

(A13) $$\begin{array}{ccc} & & \;{\hat{d}}_{i,k-1}=P_{i,k}^{dd}{\left(E_{k-1}\right)}^{T}{\left(C_{i,k}\right)}^{T}{\left({\overset{˘}{R}}_{i,k}\right)}^{-1}\left(y_{i,k}-C_{i,k}{\hat{x}}_{i,k|k-1}\right), \end{array}$$

The matrices $P_{i,k}$, $P_{i,k}^{xd}$ and $P_{i,k}^{dd}$ are calculated as follows:

(A14) $$\begin{array}{ccc} & & \;P_{i,k}=P_{i,k|k-1}-P_{i,k|k-1}C_{i,k}^{T}{\left({\overset{˘}{R}}_{i,k}\right)}^{-1}C_{i,k}P_{i,k|k-1}+P_{i,k}^{xd}{\left(P_{i,k}^{dd}\right)}^{-1}{\left(P_{i,k}^{xd}\right)}^{T}, \end{array}$$

(A15) $$\begin{array}{ccc} & & \;P_{i,k}^{xd}=\left(E_{k-1}-P_{i,k|k-1}C_{i,k}^{T}{\overset{˘}{R}}_{i,k}^{-1}C_{i,k}E_{k-1}\right)P_{i,k}^{dd}, \end{array}$$

(A16) $$\begin{array}{ccc} & & \;P_{i,k}^{dd}={\left[{\left(C_{i,k}E_{k-1}\right)}^{T}{\overset{˘}{R}}_{i,k}^{-1}C_{i,k}E_{k-1}\right]}^{-1}. \end{array}$$

From Equation (A10), we can obtain

$$\begin{array}{ccc} & & \;q\left(x_{k}\right)=N\left(x_{k};{\hat{x}}_{i,k},P_{i,k}\right). \end{array}$$

Equation (A6) can then be rewritten as

(A17) $$\begin{array}{ccc} & & \;\Delta_{i,k}=\left(y_{i,k}-C_{i,k}{\hat{x}}_{i,k}\right){\left(y_{i,k}-C_{i,k}{\hat{x}}_{i,k}\right)}^{T}+C_{i,k}P_{i,k}C_{i,k}^{T}. \end{array}$$

Equations (A7), (A9), and (A12)–(A17) show that the variational parameters are coupled. Therefore, we use fixed-point iterations to solve this problem. Let the variables with superscript (l) denote the updated estimates obtained after *l* fixed-point iterations. By replacing the variables in Equations (A7), (A9), and (A12)–(A17) with their corresponding iterates at steps (l) or (l+1), we obtain Equations (12)–(20).
